# A High-Resolution Chronology of Rapid Forest Transitions following Polynesian Arrival in New Zealand

**DOI:** 10.1371/journal.pone.0111328

**Published:** 2014-11-05

**Authors:** David B. McWethy, Janet M. Wilmshurst, Cathy Whitlock, Jamie R. Wood, Matt S. McGlone

**Affiliations:** 1 Department of Earth Sciences, Montana State University, Bozeman, Montana, United States of America; 2 Landcare Research, Lincoln, New Zealand; 3 Institute on Ecosystems, Montana State University, Bozeman, Montana, United States of America; 4 School of Environment, University of Auckland, Auckland, New Zealand; Ecole Pratique des Hautes Etudes, France

## Abstract

Human-caused forest transitions are documented worldwide, especially during periods when land use by dense agriculturally-based populations intensified. However, the rate at which prehistoric human activities led to permanent deforestation is poorly resolved. In the South Island, New Zealand, the arrival of Polynesians c. 750 years ago resulted in dramatic forest loss and conversion of nearly half of native forests to open vegetation. This transformation, termed the Initial Burning Period, is documented in pollen and charcoal records, but its speed has been poorly constrained. High-resolution chronologies developed with a series of AMS radiocarbon dates from two lake sediment cores suggest the shift from forest to shrubland occurred within decades rather than centuries at drier sites. We examine two sites representing extreme examples of the magnitude of human impacts: a drier site that was inherently more vulnerable to human-set fires and a wetter, less burnable site. The astonishing rate of deforestation at the hands of small transient populations resulted from the intrinsic vulnerability of the native flora to fire and from positive feedbacks in post-fire vegetation recovery that increased landscape flammability. Spatially targeting burning in highly-flammable seral vegetation in forests rarely experiencing fire was sufficient to create an alternate fire-prone stable state. The New Zealand example illustrates how seemingly stable forest ecosystems can experience rapid and permanent conversions. Forest loss in New Zealand is among the fastest ecological transitions documented in the Holocene; yet equally rapid transitions can be expected in present-day regions wherever positive feedbacks support alternate fire-inhibiting, fire-prone stable states.

## Introduction

The composition and structure of forests worldwide are being altered by intensification and expanding land-use, changing climatic conditions and increasing incidence of large fires [1], [2]. Sudden transitions between forest, savanna and grassland biomes can be linked to feedbacks between land-use practices, climate change and disturbances such as fire [3]. Research suggests that the extent of forest cover reinforces conditions that maintain or inhibit fire (e.g., high forest cover often promotes greater fuel moisture), and consequently, the stability of forested, savanna or grassland vegetation [4]. Recent deforestation has been shown to initiate positive feedbacks replacing forested landscapes that rarely burned with fire-prone shrubland, savanna and grasslands [5]. Human-mediated forest transitions are not a new phenomenon, however. Humans have effected forest transitions for millennia through their use of fire, and widespread rapid transitions in the past are often linked to expanding human settlements and cultivation [6], [7].

New Zealand provides a prime example of a relatively recent and major prehistoric forest loss. Here, the arrival of humans c. 750 years ago [8] instigated a period of deliberate burning, termed the Initial Burning Period (IBP), that led to the loss of nearly 50% of New Zealand's forests [9], [10] and conversion of podocarp dominated forests to open shrubland vegetation. The conversion of forests in the South Island, presents a striking paradox, that extremely small populations (by any estimate – human mtDNA sequences suggest an initial founding population of between 50–100 females and a low population growth rate of c. 1% [11]) were able to instigate widespread and permanent forest transitions. Paleoenvironmental records from New Zealand suggest that ecosystem feedbacks during the initial burning period accelerated the rapid rate of forest demise and help explain this paradox [5], [9]. In this paper, we examine the rate at which relatively stable forest biomes in New Zealand were converted to open shrublands in the 13^th^ century, and how forest transitions following early human-set fires in the South Island of New Zealand compares with other regions in the world currently experiencing deforestation.

During the IBP, nearly all pollen and charcoal records derived from the drier parts of New Zealand show a distinct decline in pollen from native podocarp tree taxa (and to a lesser extent beech forest trees), an increase in pollen from seral taxa particularly bracken fern (*Pteridium esculentum*) and grasses (Poaceae), and an increase in charcoal (e.g., [9], [12], [13]. Previous results from radiocarbon dated high-resolution microscopic charcoal analyses from a network of sites across the central South Island showed a distinct increase in charcoal accumulation rates during the IBP within 200 years following Polynesian (Māori) settlement, although the increase is diachronous among sites [9]. At all but the most remote and wet sites, paleofire reconstructions suggest that repeated fires (2 or more) were responsible for the watershed conversion of native forest to a fern/scrub or grassland. [9], [10], [14]. However, the age-depth chronologies described in [9], [14] were based on a small number of radiocarbon dates selected prior to and after the IBP, and consequently, the rate of forest loss remains poorly constrained.

**Figure 1:**
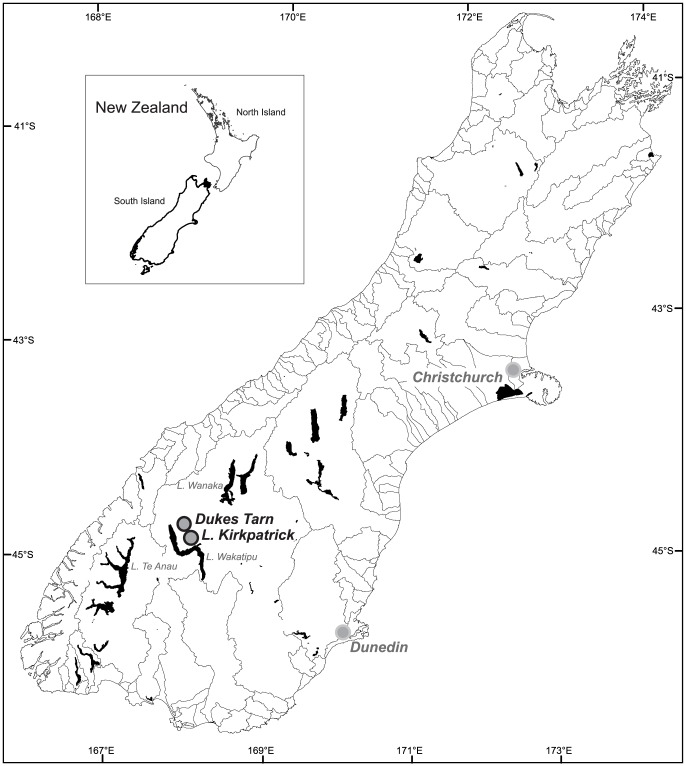
Location of study sites, South Island, New Zealand.

To precisely determine the rate at which prehistoric forest transitions occurred following initial human arrival in the South Island, New Zealand, we developed high-resolution reconstructions of vegetation (pollen) and fire (macroscopic charcoal) from radiocarbon dated lake-sediment records from two small, closed-basin lakes, Lake Kirkpatrick and Dukes Tarn (Fig. 1). We specifically choose two sites that represent different vulnerabilities to human-set fires: one drier lowland site and a second, wetter high-elevation site. The chronology of these reconstructions and rates-of-change calculations are based on a stratigraphic series of Accelerator Mass Spectrometry (AMS) radiocarbon dates from the sediment cores (Table S1).

## Results

**Figure 2:**
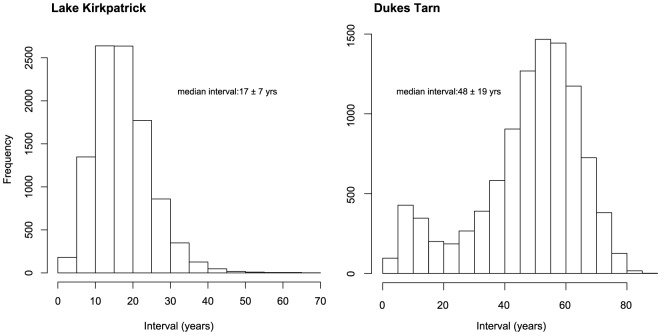
Bchron posterior frequency distribution of the interval (yrs) associated with forest transitions at Lake Kirkpatrick and Dukes Tarn, South Island, New Zealand. The interval associated with forest transitions was identified using two criteria: 1) visible change in core lithology, and 2) a rapid decline in pollen percentages of native forest taxa and a coincident increase in pollen percentages of open vegetation taxa.

**Figure 3:**
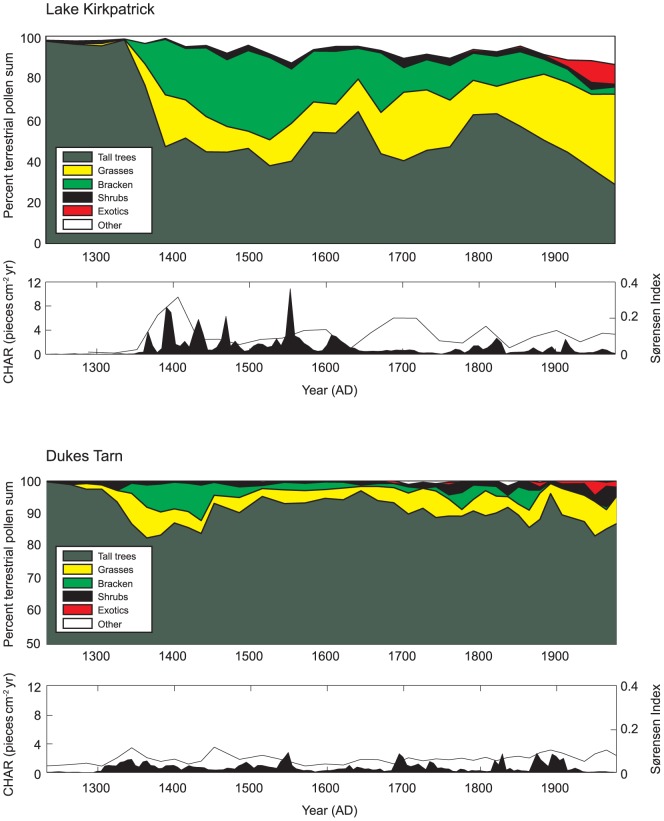
Statistical analysis of vegetation change and rate of vegetation change (based on Sørensen's similarity index) and changes in fire activity (based on CHAR, charcoal particles cm^−2^ yr^−1^, and fire event determination), Lake Kirkpatrick and Dukes Tarn. Colored panels show change in percent of total terrestrial pollen percentages for native trees and disturbance-associated taxa (e.g., Poaceae, *Pteridium*) and non-native taxa (Pinaceae, *Rumex*, *Taraxacum*-type) introduced by Europeans for Lake Kirkpatrick (top) and Dukes Tarn (bottom). Black and white panels show charcoal accumulation rates and Sørensen's distance between each pollen sample and the next oldest pollen sample.

Chronology model results were highly convergent, suggesting forest transitions occurred within decades of the first human-set fires (Figs. S1–S5). Significant (>15% decline in native taxa) forest loss occurred within 17 yrs (SD = 7) at Lake Kirkpatrick and 48 yrs (SD = 19) at Dukes Tarn (Fig. 2). At Lake Kirkpatrick, the IBP was punctuated by two fire episodes at c. AD 1367 and AD 1391 (Fig. 3, Bchron chronology). Following the initial increase in charcoal accumulation rates (CHAR; particles cm^−2^ yr^−1^), fire episodes occurred periodically (every 50–100 yrs) until c. AD 1600 when fire activity decreased. A second increase in fire activity coincided with European arrival ca. AD 1800. Vegetation assemblages changed dramatically following the first fires associated with human arrival. Native trees (e.g., *Nothofagus menziesii*, Nothofagus spp., *Podocarpus* spp. and *Prumnopitys* spp.) declined from 99 to 47% of the total terrestrial pollen percentage from 1331 to 1391 yr AD, whereas disturbance related-taxa (e.g. Poaceae and *Pteridium*) increased from up to 25 and 27%, respectively, over the same period (see Fig. S6 for additional pollen information). Pollen of native trees increased slightly to 64% at c. 1642 yr AD at the expense of grass pollen. Tree pollen percentages then declined, recovered again at c. 1792 yr AD before declining to <30% in the late 20^th^ century. Pollen of exotic taxa (Pinaceae, *Rumex* spp. and *Taraxacum* spp.) reached 2% by the mid 19^th^ century and increased to >10% at c. 1947 yr AD.

**Figure 4:**
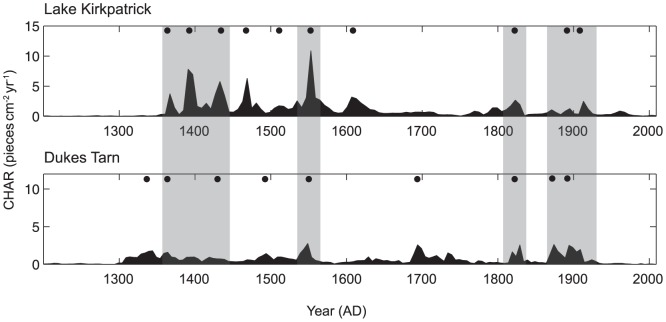
Variation in CHAR (charcoal particles cm^−2^ yr^−1^), and peak identification for Lake Kirkpatrick and Dukes Tarn. Black circles identify peaks in CHAR that are statistically significant from background variation in CHAR and are thus likely to be local fires (within 1–3 kms of lake). Shaded areas highlight time periods when fire events were recorded at both lakes.

At Dukes Tarn, charcoal data register initial fire events at c. AD 1337, and 1387, and the pollen data indicate that forests recovered within decades even though fire events occurred periodically (once every 50–100 yrs) from the IBP until the last century (Fig. 3). Pollen of some native trees (*Nothofagus fuscospora* type) collectively declined from 97 to 83% whereas disturbance-related taxa, notably Poaceae and *Pteridium*, increased from 1 to 9% and 0 to 7% respectively during the interval from c. AD 1355 to 1414 (see Fig. S7 and [9] for additional pollen information). In contrast to Lake Kirkpatrick, pollen of native trees increased to 94% by c. AD 1502 at the expense of Poaceae, then fluctuated between 85–94%, and accounted for 97% at c. 1694 yr AD (back to pre-deforestation levels). Tree percentages decreased to 83% in the last century. Non-native plant taxa (Pinaceae, *Rumex* spp. and *Taraxacum* spp.) account for >1% of the pollen at c. 1928 yr AD and 5% at c. 1980 yr AD before declining to <1% in recent decades. Fires that were recorded during the same time period at both sites (Fig. 4) likely represent a pattern of burning associated with humans moving through the landscape to enhance resource conditions and facilitate travel to nearby greenstone quarries [15] and could have been facilitated by extreme fire weather (e.g., hot, dry, windy conditions). Compared to Lake Kirkpatrick where forest transitions led to open vegetation that persists to the present, native vegetation at Duke's Tarn experienced relatively minor modification and showed partial recovery within several decades of the first IBP fires and full recovery 350 years after the IBP. Post IBP fires at Duke's Tarn seem to have had less of a lasting impact on the long-term structure and composition of vegetation.

Statistical analyses of rates of change reflect the rapid transition from native trees to grasses and bracken, evident in changes in pollen percentages (Fig. 3). Sørensen's index quantifying dissimilarity in pollen taxa prior to and during the IBP shows sharp increases in the dissimilarity in vegetation composition and dominance, with transitions in vegetation taxa peaking during the IBP at both Lake Kirkpatrick and Dukes Tarn. The Sørensen's index (dissimilarity between each sample and the previous sample) was greatest at c. 1363 yr AD (10^−2^) at Lake Kirkpatrick and at c. 1375 AD (10^−2^) at Dukes Tarn, coinciding with the first IBP fires at both sites. Dissimilarity between pre-human vegetation assemblages and post-IBP vegetation taxa increased from present to the pre-IBP period at both sites but was much more pronounced at Lake Kirkpatrick where dramatic vegetation transitions from forest to open shrublands persisted until present. Changes in vegetation assemblages at Dukes Tarn are characterized by an initial forest loss, a late recovery of native trees, followed by an increase in grasses and the presence of introduced taxa associated with European colonization.

## Discussion

Human-caused forest transitions are documented worldwide, especially during periods when land use by dense agriculturally-based populations intensified [6], [16], [17]. However, the rate at which prehistoric human activities led to a persistent biome shift is commonly poorly resolved [18]. In the South Island of New Zealand, the arrival of Polynesians resulted in dramatic forest loss, with conversion of nearly half of native forests to open vegetation by a small number of sparsely distributed populations [10]. Paleoenvironmental records suggest that this conversion occurred within decades to centuries of human arrival but the speed at which site conversion took place once burning commenced has been unclear until now. Chronological models (Bchron, Oxcal and MCAgeDepth) that explicitly estimate joint uncertainty of all radiocarbon calibration dates suggest that the forest loss during IBP was rapid, occurring in 10–24 years at the drier site, Lake Kirkpatrick (median  = 17) and 29–67 years at the wetter site, Dukes Tarn (median  = 48). The speed at which vegetation conversion occurred is likely even more rapid than estimates based on age-depth chronologies alone because they incorporate joint uncertainty originating from both AMS radiocarbon dating and radiocarbon calibration distributions. Despite inherent limitations in calculating the rate of past vegetation change from lake-sediment records, forest loss in New Zealand was rapid by any measure. These rates of deforestation parallel those made for the eastern North Island where dry, lowland podocarp forests transitioned to bracken fern shrubland within decades of the first human-set fires whereas loss of more mesic, upland forests took a century or longer [19].

For most of the South Island, fire impacts on forested ecosystems were ecologically insignificant throughout the Holocene, in that changes in the structure and composition of vegetation were poorly related to the occurrence of fire [20]. The widespread and mostly one-way shift from forest to open shrubland that occurred with the arrival of Polynesians, and then following European arrival, was unprecedented in the Holocene, even after extreme disturbance events (e.g., large volcanic eruptions). This was the case for Lake Kirkpatrick and many other sites throughout similarly dry forests of eastern New Zealand including the eastern North Island [21]. The higher-elevation site, Dukes Tarn, experienced equally rapid vegetation change, but declines in forest taxa at this site were less pronounced. This is most likely because this site is wetter and because the deeply-incised forested drainage basin south of the tarn has provided an ongoing refuge from fire, contributing significant wind-transported pollen to the tarn sediments. Differences in the extent to which fire activity and vegetation changed following human arrival at Lake Kirkpatrick and Dukes Tarn may be explained by variation in the intensity of human activity at these two sites; the most parsimonious explanation, however, centers on differences in rainfall and inferred fuel moisture at these two sites and is supported by previous results comparing a network of sites from the South Island ([9]). Paleofire records at even wetter sites (>1600 mm/yr) near or west of the Southern Alps of the South Island indicate only limited fire activity and minor associated vegetation change [9], [22]. Similarly, along the western coast of the North Island moist conditions limited the extent to which Māori were able to burn forests [23]. Here, Māori gardens were established by burning small patches of forests but the scale of forest clearing was insignificant compared to deforestation that occurred in drier regions of the North and South Island sites [23]. What is surprising is the extent and rapid rate of forest clearance that occurred in the South Island where prehistoric human populations were at their lowest compared to the North Island, and cultivation was limited to a few coastal sites north of latitude 44° South.

Dynamic Global Vegetation Models that estimate rates of prehistoric deforestation at a regional to global scale suggest that the extent and pattern of preindustrial deforestation in most regions was strongly linked to population densities, inherent landscape productivity and lifestyles and technological innovation [16], [17]. Estimates of population density and reconstructions of environmental change in these models are derived from archeological and paleoenvironmental records, and in most regions, rates of deforestation are poorly resolved. Our high-resolution chronology of environmental change from the South Island, New Zealand indicates rapid forest transitions occurred during the IBP during a time of exceedingly low population numbers and limited use of technological innovation (i.e., intensive cultivation). Why then were New Zealand's forests particularly vulnerable to fire? The lowland evergreen conifer/angiosperm forests were characterized as being both fire sensitive and ignition limited [20]. Low- to mid-elevation forests experiencing moderate to low levels of annual rainfall (300–1600 mm/yr) persisted for millennia simply because ignitions were rare and fuels were typically wet [20], [24]. It was only when human-set fires expanded the proportion of flammable early-seral vegetation on the landscape that widespread forest transitions were possible [5]. Positive feedbacks between the fire sensitivity of the vegetation, introduction of human-set ignitions, and changes in landscape flammability with the spread of early-seral vegetation offer an explanation for these rapid transitions. Perry et al. [5], [22] provide empirical support for these feedbacks, showing that dry, low- to mid-elevation evergreen conifer/angiosperm forests in the South Island New Zealand were vulnerable to fire, and that targeted burning created positive feedbacks where burning of forest resulted in more flammable seral vegetation which was more vulnerable to ignitions. The model estimated that conversion of the forest to shrubland could have occurred in 50–200 years if fires were targeted both spatially (in highly-flammable early-seral vegetation) and temporally (every few decades to maintain early-seral vegetation).

Forest transitions occurring at present in a number of regions around the world bring new attention to conditions and feedbacks that drive rapid transitions between seemingly stable states [25]–[28]. Analyzing global tree cover and fire activity data, Staver et al. [4] show that mesic landscapes (>2500 mm/y rainfall) with >60% tree cover are resistant to changes in fire activity and biome shifts, whereas areas with intermediate levels of rainfall (1000–2500 mm/y) and <50% tree cover are vulnerable to rapid transitions to savanna. Hirota et al. [29] suggest that landscapes with intermediate levels of tree cover are extremely rare because the interaction between climate, edaphic conditions and fire activity interact to attract vegetation to relatively stable states of higher (forest) and lower (savanna) levels of tree cover. A number of mechanisms create positive feedbacks that trigger and maintain these multiple stable states. Landscapes with high canopy cover of mature forests are resilient to increased fire activity because they maintain fuel moisture levels that suppress fire and often lack continuous understory fuels for carrying fire [3]. Dry landscapes reinforce open conditions by supporting low fuel moisture and species with plant functional traits that promote fire.

A consequence of these attracting states is the potential for human activities to reduce forest density and/or canopy cover past a tipping point where they become increasingly vulnerable to rapid transitions to more fire-prone states [30]. This has been shown to be particularly true for forests where flammability decreases with forest age. Kitzberger et al. [3] demonstrate that even small increases in the frequency of ignitions and/or climate variability in these forests can result in dramatic shifts in fire regimes at large scales. The New Zealand example suggests that targeted ignitions alone where sufficient to initiate feedbacks promoting high landscape flammability and increased fire activity.

Today, the vulnerability of temperate forests to rapid transitions is evident in a number of regions. For example, alterations to fuel and microclimate conditions following logging activities in evergreen forests of the northwestern US increase post-fire flammability, ultimately increasing fire severity such that forest recovery is delayed or non-existent [31], [32]. Lindenmayer et al. [27] describe a similar feedback in mountain ash forests in Victoria, Australia where logging activity results in an abundance of fine fuels, slash and more flammable early-seral vegetation. While fire occurred in these forests in the past, it was typically infrequent, and eventually followed by conditions that promoted robust recruitment of mountain ash seedlings. In recent decades, the increase in fine fuels, slash and persistently drier microclimatic conditions following logging activity and subsequent increased fire activity interact to create these new fire-prone ‘landscape traps’ [27]. Likewise, Cochrane et al. [33] demonstrate that previously burned forests in Amazonia are highly vulnerable to subsequent fires that further promote more open, highly-flammable vegetation. In southern South America, the combination of increased anthropogenic fires and herbivory of native seedlings by introduced species is rapidly facilitating the replacement of mesic *Nothofagus* forests with fire-prone shrubs [34]. On Great Barrier Island North Island, New Zealand, Perry et al. [35] found that conversion of mesic broadleaf forests to shrublands initiated positive feedbacks in which an increase in fire activity not only reinforced the spread and persistence of highly flammable early-seral woody vegetation but also promoted flammable invasive species and degraded soil conditions, especially on drier aspects. Lacking fire, these forests would likely recover but Perry et al. [35] observe that, along with a continued loss of topsoil, seed trees and dispersal agents (e.g. avian frugivores) are becoming increasingly rare such that forest recovery becomes ever more improbable. These recent forest transitions demonstrate the strong influence of human-initiated positive feedbacks that increase landscape flammability and highlight the inherent vulnerability of seemingly stable temperate forests to rapid biome and fire regime shifts. Widespread forest transitions are predicted for the future in regions with similar age-flammability and low-ignition characteristics [36].

## Conclusions

The timing of an abrupt change from forest to open shrubland following human-set fires at two sites in the South Island of New Zealand is well-constrained in sediment cores using high-resolution chronology models, suggesting widespread deforestation occurred within decades of the first anthropogenic fires in the most vulnerable drier regions. The fire history of New Zealand is an example of how a seemingly stable ecosystem can pass natural tipping points and experience persistent transformations. The rate at which anthropogenic forest loss occurred was rapid, taking place within decades following the initial arrival of a small founding population. The rapidity of the response was partly facilitated by positive feedbacks created by the introduction of a new and frequent ignition source by humans. In addition, the dominant forest taxa lacked natural adaptations to fire (e.g., serotiny, resprouting) which made them highly vulnerable to increased ignitions [24]. The newly created fire-prone landscapes persisted even when fire activity decreased after the IBP. These shrublands will likely be maintained for centuries if not millennia unless fire is removed from the system for long periods of time (>100 years). As with temperate forests experiencing similar transitions today, simply removing fire from New Zealand landscapes could eventually lead to forest recovery. However, recent examples suggest that a confluence of factors that degrade biophysical conditions, impede seedling regeneration, remove source populations and dispersal agents and introduce fire-adapted weedy species will continue to hinder native forest recovery in New Zealand and elsewhere. Even in the absence of fire, management of landscapes to promote open vegetation, agriculture, and exotic forestry plantations promises to reinforce conditions that prevent regeneration of native species. The rapid rate of forest transitions witnessed in New Zealand 750 years ago portends potentially rapid transitions in biomass-rich regions experiencing similar land-use-disturbance interactions and feedbacks and highlights challenges to native forest recovery.

## Materials and Methods

### Study Sites

The study regions lie in the southeastern hill country and southern alpine region of the South Island of New Zealand, an area that ranges between 250 and 1900 m elevation (Fig. 1). The climate is cool year-round and moderately wet with most of the precipitation occurring in winter. There is a strong steep east/west gradient both in precipitation and elevation. During the Holocene the vegetation of the region was dominated by closed-canopy broadleaf evergreen forests [20]. Natural fires occurred in areas of forest in the dry southeastern region of New Zealand but were characterized by long intervals between fires [37]. The original forest extent and composition are inferred from pollen records, isolated surviving forest stands and remnant wood [38], [39]. Permission to conduct fieldwork at the two study sites was granted by the New Zealand Department of Conservation (DOC).

#### Lake Kirkpatrick (lat. 45.03°S, long. 168.57°E; 567 masl)

The Lake Wakatipu catchment is a mid-elevation (570 masl) site receiving moderate levels of rainfall (1077 mm/yr) and was part of a trade route for highly prized greenstone that the indigenous population (Māori) used and traded [15]. Lake Kirkpatrick lies within the Lake Wakatipu catchment. The small lake (∼3.0 ha) is surrounded by pastureland with browntop (*Agrostis capillaris*), Poa spp. and other introduced grasses, clover (*Trifolium* spp.), *Hypericum* spp.; pine plantations (*Pinus radiata*); and small areas of native silver beech forest (*Nothofagus menziesii*).

#### Dukes Tarn (lat. 44.96°S, long. 168.49°E; 825 masl)

Dukes Tarn is approximately 9.5 km northwest of Lake Kirkpatrick at 830 masl and also located within the Lake Wakatipu catchment. Dukes Tarn (∼1.5 ha) receives more annual rainfall (1340 mm/yr) than Lake Kirkpatrick and vegetation surrounding the lake consists of patches of black beech (*Nothofagus solandri*), small trees and shrubs (e.g., *Coprosma* spp. *Phyllocladus, Dracophyllum longifolium, Hebe* spp., *Gaultheria* spp.), native tussock (*Chionochloa* spp.) and introduced pasture grasses (*Festuca* spp.). Deeply-incised drainages supporting native forest remnants border Dukes Tarn to the south whereas open vegetation surrounds the tarn on rolling terrain to the north.

### Lake Sediment Cores

Lake sediment cores were obtained from Lake Kirkpatrick and Dukes Tarn using a polycarbonate tube (Klien core). Cores were split at University of Minnesota's LacCore facility for charcoal and pollen analysis. A 195 cm sediment core was retrieved from Lake Kirkpatrick in 2009 and a 173 cm sediment core was retrieved from Dukes Tarn in 2008. Core lithology for Lake Kirkpatrick consisted of light green gyttja 0–27 cm (depth below surface), gray silty clay 27–29 cm, dark green gyttja 29–105 cm, light gray silty clay 105–115 cm, and light green gyttja 115–195 cm. Core lithology for Dukes Tarn consisted of light green gyttja 0–42 cm, silty clay 42–43 cm, light green gyttja 43–128 cm, dark silty clay with charcoal 128–136 cm, and light green gyttja 138–173 cm.

### Reconstruction of Vegetation and Fire Histories

Pollen analysis followed the preparation methods of Moore et al. [40]. Pollen counts exceeded 250 terrestrial pollen grains and bracken (*Pteridium*) spores. Percentage calculations were based on this terrestrial sum (excluding other ground ferns and tree fern spores as they tend to be over-represented). We followed the protocol of McGlone and Wilmshurst [10] to detect initial human impact on the vegetation and grouped pollen taxa into tall forest taxa (predominantly *Fuscospora* spp. and podocarps); disturbance-related taxa including ferns (*Pteridium esculentum*), *Coriaria* spp. and grasses (Poaceae); and introduced taxa, including Pinaceae and *Rumex* spp. Remaining taxa, mostly herbaceous species, were labeled “other”. These groupings were used to identify the transition from closed forest to open shrubland and grassland.

High-resolution charcoal analysis followed methods of Whitlock and Larsen [41]. Charcoal particles greater (>125 µm in diameter) were examined to reconstruct local fire events based on changes in charcoal accumulation rates (CHAR; particles cm^−2^ yr^−1^). Decomposition of the charcoal time series employed a Gaussian mixture model to identify the mean and variance of the background CHAR (BCHAR) distribution [42]. The 99^th^ percentile of this distribution is defined as the threshold value separating peak fire events from “noise”. Significant charcoal peaks, determined by CHAR values greater than a locally-defined threshold value, identified specific fire events. Because each 1-cm vertical interval in the lakes encompasses 1–10 years at both sites, a charcoal peak is assumed to represent one or more fires occurring within the time span of a charcoal peak. The statistical significance of each peak was evaluated by comparing the original charcoal counts against the values in samples occurring 35 years before the peak. If the maximum count of a peak had a >5% chance of coming from the same Poisson-distributed population as the minimum charcoal count within the preceding 35 years, then a “peak” was not identified. Fire size and/or intensity were inferred from the magnitude of individual charcoal peaks (particles cm^−2^ yr^−1^) [43].

### Chronology Models

We obtained 22 radiocarbon dates at two sites to develop high-resolution chronologies (Table S1). Radiocarbon dates were obtained from macrofossils found during the interval of the first IBP fires where changes in charcoal accumulation rates and pollen percentages indicate dynamic changes in fire activity and vegetation. The chronology of these reconstructions and rates-of-change calculations are based on a stratigraphic series of AMS radiocarbon dates from the sediment cores. We incorporated this suite of radiocarbon dates into three readily accessible chronology models designed to estimate lake-sediment age-depth chronologies and chronological uncertainty: Bchron [44], Oxcal [45] and McAgeDepth [43]. Model outputs were highly convergent for all three models (Figs. S1–S5). We highlight results from Bchron because the chronology model has an environment event analysis function which explicitly estimates the duration of the interval at which significant environmental events occurred (Fig. 2).

Accelerator mass spectrometry (AMS) ^14^C dates obtained on twig charcoal and terrestrial plant macrofossils, provided the chronology for precisely estimating rates of past environmental change. Thirteen radiocarbon dates were obtained from Lake Kirkpatrick and nine from Dukes Tarn. Eleven radiocarbon dates were obtained from across 20 centimeters of sediment where charcoal and pollen percentages identify the IBP at Lake Kirkpatrick and six radiocarbon dates were obtained across the comparable 10-centimeter interval at Dukes Tarn. We used this set of radiocarbon dates in a suite of age-depth chronology models to estimate the duration at which forest transitions occurred.

To derive age-depth chronologies we used three statistical age-depth chronology models: Bchron [44], Oxcal [45] and McAgeDepth [43] which all address two primary types of uncertainty: uncertainty in radiocarbon dates and their calibration, and variability in the sedimentation process itself. These models share a number of features that are desirable for developing a continuous age-depth chronology for sediment cores including, monotonicity (deeper sediments are likely older), variable sedimentation (rates are assumed to be variable), probabilistic interpolation based on uncertainty associated with calibrated probability distributions (posterior samples or chronologies are based on joint estimates of uncertainty of all dates), and increased uncertainty at depths far from radiocarbon-dated material [46]. Each model uses a Markov-Chain Monte-Carlo algorithm to estimate model parameters, which provides a distribution of the chronologies that are most likely given all radiocarbon-dated probability distributions. Most importantly, they explicitly account for and estimate joint uncertainty of all of the radiocarbon dates and consider the probability density functions of calibrated age ranges in chronology interpolation. All high-resolution chronology models achieved convergence and identified no outliers at Dukes Tarn, but two outliers at Lake Kirkpatrick (macrofossil dated material at depths 110 and 111 cm), which were subsequently left out of modeled chronologies. The radiocarbon material at these two depths were identified as having low mass during AMS analyses (KCCAMS lab), which likely increased their age uncertainty and inaccuracy.

### Rate of vegetation change

We used the Sørensen's similarity statistic [47] to assess the rate of vegetation change between pollen samples. Sørensen's index, similar to the Bray-Curtis dissimilarity [48] is a similarity index that measures compositional similarity between two sites, or in this case, compositional similarity in pollen taxa at two depths where pollen percentages are measured. Sørensen's distance statistic is calculated between each pollen sample and the preceding pollen sample providing a statistical measure of vegetation change over time.

## Supporting Information

Figure S1 **Comparison of chronology model estimates (circles and bars represent median and 95% Confidence Intervals) for the interval associated with forest transitions that followed the first human-set fires, Lake Kirkpatrick and Dukes Tarn, South Island, New Zealand.** Median (and 95% confidence estimate ranges) for the interval associated with forest transitions for Lake Kirkpatrick were 17 yrs (3–13, Bchron), 27 yrs (7–47, MCAgeDepth), and 31 yrs (16–46, Oxcal). Median and 95% confidence estimate ranges (in parentheses) for the interval associated with forest transitions for Dukes Tarn were 47 yrs (35–59, Bchron), 44 yrs (24–64, MCAgeDepth), and 25 yrs (14–36, Oxcal).
(EPS)

Figure S2 **MCAgeDepth age depth model for Lake Kirkpatrick with sedimentation rates and sample resolution.** Chronologies developed using a weighted cubic smoothing spline which takes into account the number and uncertainty of age estimates. Gray shading represent 95% confidence intervals reflect the combined uncertainty of all age estimates in a model and are derived from 1000 bootstrapped chronologies.
(EPS)

Figure S3 **MCAgeDepth age depth model for Dukes Tarn with sedimentation rates and sample resolution.** Chronologies developed using a weighted cubic smoothing spline which takes into account the number and uncertainty of age estimates. Gray shading represent 95% confidence intervals reflect the combined uncertainty of all age estimates in a model and are derived from 1000 bootstrapped chronologies.
(EPS)

Figure S4 **Oxcal age-depth model for Lake Kirkpatrick using the P_Sequence deposition model which assumes deposition to be random (** ***k*** **parameter set at 1 – which assumes a postulated event spacing of approximately 1 cm).** Light grey distributions represent likelihoods for single calibrated dates and in darker grey shading represents the marginal posterior distributions which take into account the depth model; the depth model curves show envelopes for the 95% and 68% highest probability density (HPD) ranges.
(EPS)

Figure S5 **Oxcal age-depth model for Dukes Tarn using the P_Sequence deposition model which assumes deposition to be random (** ***k*** **parameter set at 1 – which assumes a postulated event spacing of approximately 1 cm).** Light grey distributions represent likelihoods for single calibrated dates and in darker grey shading represents the marginal posterior distributions which take into account the depth model; the depth model curves show envelopes for the 95% and 68% highest probability density (HPD) ranges.
(EPS)

Figure S6 **Pollen diagram for Lake Kirkpatrick, South Island, New Zealand.**
(EPS)

Figure S7 **Pollen diagram for Dukes Tarn, South Island, New Zealand.**
(EPS)

Table S1 **AMS radiocarbon dating determined age information for Lake Kirkpatrick and Dukes Tarn, New Zealand.**
(PDF)
