# Supplementary material for: A High-Resolution Chronology of Rapid Forest Transitions following Polynesian Arrival in New Zealand
Source: PLoS One. 2014 Nov 5;9(11):e111328. doi: 10.1371/journal.pone.0111328 (PMC4221023; doi:10.1371/journal.pone.0111328)
Supplement: Table S1 — AMS radiocarbon dating determined age information for Lake Kirkpatrick and Dukes Tarn, New Zealand. (PDF) [file pone.0111328.s008.pdf]

Table S1. Radiocarbon Dating Information. Summary of Conventional Radiocarbon Ages (CRA) in years Before Present (i.e., before 1950 AD) and CRA error (± yrs), for radiocarbon dated macrofossils Lake Kirkpatrick and Dukes Tarn, South Island, New Zealand.

Lab ID	Site (year core collected)	Material dated	Depth (cm)	CRA	CRA error
KCCAMS-55542	Dukes Tarn (2008)	leaf	33-34	210	20
KCCAMS-55541	Dukes Tarn (2008)	leaf	119-120	390	15
KCCAMS-102198	Dukes Tarn (2008)	twig	129-130	710	15
KCCAMS-102199	Dukes Tarn (2008)	charcoal	131-132	680	20
KCCAMS-102200	Dukes Tarn (2008)	leaf	132-133	710	30
KCCAMS-102201	Dukes Tarn (2008)	twig	135-136	700	15
KCCAMS-102202	Dukes Tarn (2008)	leaf	137-138	720	15
KCCAMS-55540	Dukes Tarn (2008)	leaf	139-140	670	15
KCCAMS-71876	Dukes Tarn (2008)	leaf	165-166	905	20
KCCAMS-102187	Lake Kirkpatrick (2009)	leaf	99-100	575	15
KCCAMS-102188	Lake Kirkpatrick (2009)	char/seed	104-105	650	30
KCCAMS-102189	Lake Kirkpatrick (2009)	char	108-109	680	20
KCCAMS-102190	Lake Kirkpatrick (2009)	leaf	109-110	640	15
KCCAMS-102191	Lake Kirkpatrick (2009)	char	110-111	555	20
KCCAMS-102192	Lake Kirkpatrick (2009)	twig	111-112	750	15
KCCAMS-102193	Lake Kirkpatrick (2009)	twig	112-113	695	20
KCCAMS-102194	Lake Kirkpatrick (2009)	wood	113-114	635	15
KCCAMS-102195	Lake Kirkpatrick (2009)	plant part	114-115	670	30
KCCAMS-102196	Lake Kirkpatrick (2009)	leaf	118-119	670	20
KCCAMS-71877	Lake Kirkpatrick (2009)	woody material	119-120	645	15
KCCAMS-102197	Lake Kirkpatrick (2009)	leaf	121-122	710	15
KCCAMS-71878	Lake Kirkpatrick (2009)	leaf	191-192	1625	15
